# Novelty detection and orienting: effects on skin conductance and heart rate

**DOI:** 10.1007/s00426-022-01735-2

**Published:** 2022-09-15

**Authors:** Heinz Zimmer, Fabian Richter

**Affiliations:** 1 Department of Psychology, University of Cologne, 50931 Cologne, Germany; 2 Department of Psychosomatic Medicine, German Heart Center Berlin, 13353 Berlin, Germany

## Abstract

In a repetition-change paradigm it was explored whether the skin conductance response (SCR) and the heart rate (HR) response similarly reflect involuntary and voluntary orienting. Seven orienting stimuli, consisting of six contextually novel stimuli and one novel change, were presented to 144 participants. In order to evoke voluntary orienting, the signal value of the contextually novel stimuli was manipulated by task instruction. Results suggest that the SCR is a manifestation of the involuntary orienting response (iOR). The HR, however, showed a non-uniform pattern of response and turned out to be susceptible to voluntary orienting. While it responded to the last orienting stimulus, the novel change, with a clear-cut deceleration, the response to the first orienting stimulus had a polyphase structure and was sensitive to repetition and signal value. The HR response is, thus, of limited value as an indicator of the iOR because of its striking susceptibility to voluntary orienting.

## Introduction

Psychological research is, whenever possible, guided by theory. However, stringent hypothesis testing or reasonable practical application of a theory is often reliant on good indicators of processes or latent variables mentioned in the theory. In search of such indicators, a great deal of work has been invested by the international research community. But even in a field of research as fundamental as the investigation of orienting, it has not yet been convincingly established empirically whether two well-known autonomic responses to novel stimuli—the skin conductance response (SCR) and the heart rate (HR) response—indicate the brain activity of the orienting mechanism in an unambiguous and comparable manner. This mechanism was introduced to psychology years ago by an influential theory of orienting (cf. Sokolov, 1960, 1963, 1975, 1990) and it is still crucial to the understanding of human behaviour and learning. For this reason, we address the question of whether these two peripheral responses similarly reflect *novelty detection*, the theoretical basis or key process for the triggering of the *involuntary orienting response* (OR). In this vein, we are interested in whether both of them are valid indicators of novelty detection and thus indicate involuntary orienting straightforwardly and independent of voluntary orienting.

*Novelty detection*, i.e., detection of a contextually or actually novel event, is typically linked to avoiding dangers, adapting to environmental changes and improving one’s knowledge. A novel stimulus by definition evokes the *involuntary* OR, an evolutionarily arisen response mechanism that ensures that processing resources are automatically focussed on unknown events to incorporate them into an enhanced representation of the world (Lynn, 1966; Pavlov, 1927). Novelty is also considered to trigger involuntary orienting because of an inherent and thus unconditioned signal value—that is, unfamiliar stimuli point to potential but vital consequences that must be attended to (Campbell et al., 1997). As soon as the OR releasing stimulus occurs repeatedly without any consequences it becomes familiar and its relevance for improvement of perception and further processing decreases rapidly. This selective adaptation is one of the most elementary forms of learning and memory (Ranganath & Rainer, 2003).

*Involuntary orienting* is thus driven by novelty detection and inevitable coupled with an automatic or passive shift of attention because attentional control is in a sense captured by novelty (Johnston et al., 1990). Nevertheless, involuntary orienting may subsequently be replaced by *voluntary orienting* as soon as attention is controlled by recognising clues for a significant event that is relevant to goals or actions. The conception of two different operating or control modes underlying different orienting activities originates in the classical distinction between two basic *varieties of attention*: (i) passive, non-voluntary, effortless attention; and (ii) active and voluntary attention (James, 1890). In other words, it is taken for granted that selective processing can be performed passively and actively (Johnston & Dark, 1986, p. 63). It is, however, important to bear in mind that attention (selection) is not orienting, and that a different attentional control as well as its triggering is only one defining feature of the differentiation between involuntary and voluntary orienting. Other distinctive features rest upon the involved processing operations and, what still has to be proved (see below), their consequences in peripheral response systems.

Thus, orienting is thought to be a brain response to novelty, significance, as well as an amalgam of both (e.g., Bernstein, 1969, 1979; Dawson et al., 1989; Maltzman, 1979, 1990; Pendery & Maltzman, 1977; Sokolov, 1963, 1990). Some of the significant stimuli already have an inherent biological signal value, others acquire their signal value by conditioning or simply by instruction.

With this in mind, our current OR paradigm—a special variant of the well-known repetition-change paradigm—was implemented to examine the effects of novelty and signal value on two presumable response manifestations of the OR, the skin conductance response and the heart rate response. To elicit an *involuntary orienting response*, a pure novelty OR, an OR associated with the involuntary and passive shift of attention, a contextually salient and unexpectedly occurring novel—and consequently unfamiliar—sound (change condition) was presented after a series of six familiar auditory stimuli. These familiar stimuli had a crucial feature in common, they belonged to a conceptual category (conceptual repetition condition): all of them were one-digit numerals. Moreover, independent of familiarity, the first stimulus in this series, in particular, can be regarded as contextually novel within the context of the particular experimental setting. This special event is thus expected to serve as a stimulant to another kind of novelty OR, an OR to *contextual novelty*. Beyond that, the present paradigm differs from classical variants especially by assigning a special signal value to some of the familiar stimuli that promotes *voluntary orienting*. *Signal value* was established by an instruction that prompted participants to pay close attention to three out of the six one-digit numerals (targets) and their immediate consequences. This instruction takes account of the hypothesis that the “significance contribution to the OR is achieved via frontal lobe activation of voluntary attention” (Sokolov, 1990, p. 99), and it aims to emulate exploration behaviour, a striking element of orienting outside the laboratory world (Berlyne, 1966; Daffner et al., 1998, 2003).

In a typical OR paradigm an exemplary OR manifestation is expected to show the unique features of the involuntary OR, *response habituation* and *response recovery* (Siddle, 1991; Sokolov, 1963). Response habituation is the expected and usually exponential amplitude decline with repeated stimulation. Response recovery is the subsequent increase in amplitude after a distinguishable change in stimulation. If this recovery does occur, the preceding response decline can be interpreted as a sign of a *selective* central nervous system (CNS) inhibition process (e.g., Sokolov, 1960, 1963; Voronin & Sokolov, 1960), because a generalised CNS process such as fatigue is incommensurate with response recovery. This selective inhibition process is usually called (inferred process of) habituation.

By definition, every unequivocal manifestation of Sokolov’s involuntary OR (e.g., 1963) needs to show response habituation *and* response recovery. Sokolov (e.g., 1963, 1975) even emphasised that the effects of repetition and change should appear in *all* OR manifestations *in a comparable manner*.

To explain the selective nature of habituation, Sokolov (1963) introduced the concept of a “neuronal model of the stimulus”. Each occurrence of a redundant event increases the precision of this model and thus the (selective) inhibition of the OR, while the occurrence of a discrepancy, i.e., of a mismatch between stimulus and model, will again trigger an OR. The greater the mismatch, the larger will be the response recovery (Voronin & Sokolov, 1960).

Referring to the question of whether the HR response is a manifestation of the involuntary OR pending issues mainly stem from conflicting empirical findings. Although, at times the HR exemplarily shows response recovery indicating (renewed) novelty processing (e.g., Bohlin et al., 1981; Simons et al., 1987, experiment II; Turpin et al., 1999; Vossel & Zimmer, 1989a, 1992; Zimmer, 2002), the proofs about response habituation under pure stimulus repetition have been inconsistent (e.g., Barry, 1986; MacDonald et al., 2015; Simons et al., 1987; Turpin et al., 1999; Vossel & Zimmer, 1989a). For a certain HR response—a distinctive deceleration—Zimmer (2002) even found response habituation *as well as* response recovery. However, considering the empirical contradictions, a clear link between the OR and HR deceleration is highly questionable. Possible HR modulations, which could result from various processing operations in different phases of processing, also impede the assumption of a simple relation (see below).

Resting upon the observation that recovery of the cardiac response is occasionally (a) a rapidly developing, (b) very pronounced and (c) relatively long-lasting slowing of the heartbeat frequency (cf. Bohlin et al., 1981; Vossel & Zimmer, 1989a, 1992; Zimmer, 2002), it may be concluded that it consists of two or three phases of HR deceleration with different starting times: (i) a brief, rapid-onset deceleration or *primary bradycardia* (Lacey & Lacey, 1980) representing stimulus registration (Graham, 1992), (ii) a *second deceleration* reflecting involuntary orienting (e.g., Graham & Clifton, 1966; Turpin, 1983), and possibly (iii) a *third deceleration* reflecting voluntary orienting activity associated with supervisory attentional control of processes involved in anticipation and preparation (Bohlin & Kjellberg, 1979; Damen & Brunia, 1987; Lacey & Lacey, 1978).

Referring to a more recent concept of preparation, findings suggest that preparation is a set of processes (Jennings & Van der Molen, 2005) and that transient heartbeat slowing is indicative of inhibitory processes necessary for an appropriate task-based preparation (Jennings & Van der Molen, 2002). A task-based HR deceleration may thus be a useful psychophysiological window into the operating principle of the supervisory attentional system (Jennings & Van der Molen, 2002, pp. 337–340).

In the transitional phase (involuntary orienting) between stimulus registration and voluntary orienting, various processes are likely to be triggered by novel stimuli. These may either (a) support slowing of the heartbeat frequency. Or, (b) depending on stimulus features (especially rise time and intensity; cf., e.g., Cook & Turpin, 1997), signal value (e.g., Öhman et al., 2000; Walter & Porges, 1976) and, most importantly, the central processing requirements associated with the particular signal value (Graham, 1992; Jennings, 1986; Lacey, 1967; Simons et al., 1998), they might drive the heart rate up even above baseline, so that the cardiac response finally has a three-phase structure.

In the case of a novel event without any task or goal relevance and of moderate intensity and rise time, one of these processes, an automatic and consequently passive *call for processing* (Öhman et al., 2000), is assumed to trigger the slowing of the heartbeat frequency. This deceleration of the HR is presumably a pure *novelty-dependent deceleration* because *novelty detection,* the theoretical CNS core component of *involuntary orienting* (e.g., Graham & Hackley, 1991; Öhman et al., 2000), is the presumed cause of this call. Duration of this “*novelty deceleration”* is assumed to be longer than the primary bradycardia and shorter than a chronologically third deceleration, which we associate with voluntary orienting. Just like the latter, a *novelty deceleration* is directly connected with reduced somatic activity and improvement of perception (cf. Öhman et al., 2000, pp. 552–556).

### Hypotheses

(A) In light of the above considerations regarding the component structure of the cardiac response and its susceptibility to various CNS processes, we hypothesize (I) that the *reaction of the heart* to our *task-irrelevant* and *definitely unknown* stimulus is a very pronounced, rapidly developing and relatively long-running HR deceleration, as conditions causing accelerative tendencies were non-existent, whereas at least two CNS causes of deceleration were given—(1) stimulus registration and, most importantly, (2) the call for processing.

In contrast to this clear-cut deceleration, we expect (II) the general cardiac response to our *familiar* stimuli to have a three-phase structure, consisting of a transient and purely stimulus-driven deceleration, followed by an acceleration and another deceleration. Regarding (a) the *task-relevant* familiar stimuli (targets), we assume that the acceleration and the ensuing deceleration are due to the particular signal value of the stimuli and thus to their demands on central processing (effortful memory processes and decision making) and active attentional processes (concerning perception and anticipation). In terms of (b) the *remaining* familiar stimuli (non-targets), the same (three-phase) response structure is expected to occur but to a lesser extent—due to lower demands on the central processing and the active attentional processes. The reason for these lower demands is that further effortful processing and supervisory attentional control are no longer necessary once a stimulus has been identified as a non-target.

However, if the cardiac response to the stimuli of our repetition-change paradigm were (merely) a pure indicator of novelty processing, it would have to be a strong and clear-cut novelty-dependent HR deceleration, behaving like an exemplary indicator of involuntary orienting.

(B) The *skin* is expected to respond to the novel change as well as to the preceding familiar stimuli in almost the same manner—an increase in conductance. This increase in skin conductance, the SCR, is expected to reflect contextual novelty (in the present case, particularly the OR to the first stimulus), actual novelty (in the present case, the recovery of the OR to the novel change), as well as the CNS process of habituation (as a result of conceptual repetition).

Specifically, conceptual repetition is expected to decrease the SCR and novelty as well as signal value (voluntary orienting) are expected to increase the SCR. In addition, response habituation, i.e., the decrease in the SCR with repeated conceptual stimulation, is expected to follow an exponential course.

No sound hypothesis can be formulated about the interactive effect of signal value and repetition on the amplitude of the SCR as well as on the component structure of the HR response, in particular because it is not clear whether voluntary orienting, as implemented in the present study, habituates.

## Methods

### Participants

Participants were 144 (40 male, 104 female) volunteers aged from 18 to 43 years (mean age: 24 years; median: 23; S.D.: 4.1). They reported no history of hearing loss or hearing difficulties and were not paid for their participation. The study was conducted in accordance with the ethical standards of the 1964 Helsinki Declaration and its later amendments. Informed consent was obtained from all participants prior to the experiment.

### Procedure and stimuli

Prior to a 6-min pre-experimental rest period, participants were truthfully informed that a sequence of six soft-spoken numbers would be presented in a little while and without further announcement or interruption. They were asked to relax without using common relaxation techniques and, apart from that, to follow their task instruction (cf. “Instruction and experimental manipulations”). Furthermore, the participants were requested not to anticipate stimuli during the physiological data recording.

**Fig. 1:**
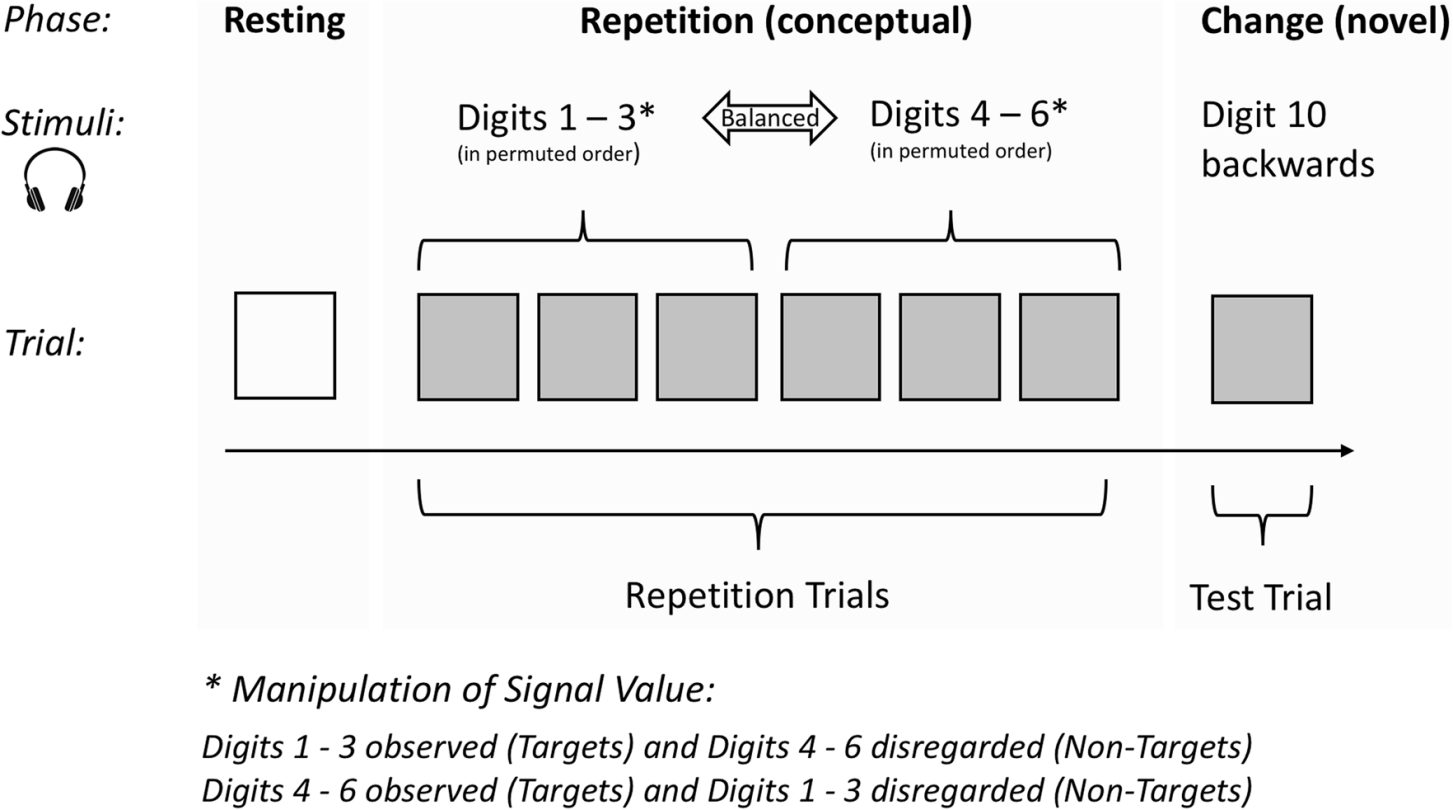
Realised auditory repetition-change paradigm. Note, each participant received a sequence of seven verbal stimuli, six soft-spoken single digits (single-digit numbers) followed by a unique sound (the digit 10 spoken backwards). The numbers were contextually novel whereas the sound was entirely novel. The sequence of numbers represents a conceptual repetition and the unique sound the change

All stimuli were digitised, equalised regarding onset latency, and played back by a commercial PC sound card. They were presented over loudspeakers, in a sequence consisting of seven stimuli, six numerals followed by a unique sound (the digit 10 presented backwards). To avoid most refractory-like effects, all stimuli were presented at long intervals (of constant 16 s). Intensity of these speech stimuli was approximately comparable and near 60 dB SPL (re: 0.0002 dynes/cm^2^) at the headrest of the participant’s chair. Due to balancing, 72 different numerical series were utilised in the study. They represent the *conceptual repetition* and were suitable for measuring *response habituation* (cf. Zimmer, 2002). In conjunction with a different task instruction (cf. “Instruction and experimental manipulations”), each of these series, but only one at a time, was presented to two participants. The 72 numerical series had one feature in common: they all consisted of the acoustically presented digits 1, 2, 3, 4, 5 and 6. The unique sound was in each case presented finally, viz. in the seventh trial (a test trial). It was used for measuring *response recovery,* which is an essential test of whether measured response habituation across the digit presentations (repetition trials) clearly indicates CNS habituation. The digits were not presented randomly across the repetition trials but in groups of three stimuli each. The digits 1, 2, 3 on the one hand, and the digits 4, 5, 6 on the other, belonged to different presentation groups—with a balanced order being realised both within and between these groups (see Fig. 1).

To make sure that the participants voluntarily focused attention on nothing but one of these presentation groups at a time, they were given the task of paying close attention to the respective numerals and neglecting the others.

### Instruction and experimental manipulations

Participants were truthfully informed that they would hear six soft-spoken numbers.

#### Within-subjects manipulation of signal value

Signal value of stimuli was experimentally manipulated by a two-part instruction: to *actively attend* some specific stimuli and their immediate consequences (“high” signal value) and to *disregard (in a passive or tolerating manner)* all the others (“low” signal value). Consequently, for each subject, a group of three stimuli (either digits 1, 2, 3 or digits 4, 5, 6) was clearly defined as targets to be attended to. These were the task-relevant stimuli (targets), whereas the remaining stimuli were non-targets because they were not to be actively attended to.

#### Content of the two-part instruction used for manipulation of signal value

One part of the instruction (promoting *voluntary* orienting) asked participants to find out whether the specified targets were (a) spoken by a *female* voice, (b) contaminated by a noise, or (c) followed by any noise in the subsequent 8 s. In fact, none of the targets were spoken by a *male* voice, superimposed by a noise, or followed by a noise.

The other part of the instruction (promoting *involuntary* orienting) asked participants to take no notice of non-targets. If a non-target would attract attention passively, involuntarily, or unintentionally, they should be unconcerned about it and stay relaxed.

#### Manipulation check

After the stimulus presentation was over, participants were invited to answer questions about the stimuli and their task at hand. All of them declared that they tried to meet the requirements. More importantly, they could reproduce key aspects of the task instruction in their own words.

#### Positioning of the targets within the series of numerals

To investigate whether it makes a difference if targets occur in early or late repetition trials (commonly also referred to as habituation trials), all targets were presented with an equal frequency across the halves of these series of trials. In this way, the between-subjects variable *target positioning* was introduced as a further experimental factor. Thus, half the participants (72) received their three targets in the first half and the others (72) in the second half of the numerical series, each of which comprised six numbers. The two levels of the between-subjects variable did not differ significantly in age, gender, social affiliation and electrodermal lability of the participants. The novel stimulus, presented in the seventh trial (*test trial*), was irrelevant to the task and not mentioned in the instruction (cf. Fig. 1).

### Physiological recording and treatment of raw data

The electrocardiogram was obtained (at 500 Hz) from two sintered Ag/AgCl electrodes filled with commercial ECG Electrode Gel. Electrodes were attached to the manubrium of the sternum and the left lower rib cage. R-waves were detected offline. Subsequently, R-R intervals were converted into heart rate (in beats per minute—bpm). The HR was then sampled on a second-by-second basis for the pre-stimulus second and the eight seconds following stimulus onset (commonly referred to as *post-stimulus seconds*) according to the formula provided by Velden and Wölk (1987; cf. also Velden & Graham, 1988).

Recording of electrodermal activity was accomplished using sintered Ag/AgCl electrodes (1 cm in diameter) filled with 0.05 M NaCl electrolyte. The electrodes were placed on the thenar and hypothenar eminences of the left hand using adhesive rings. Skin conductance (SC) was detected by a constant voltage (0.5 V) coupler (for details, cf. Zimmer & Demmel, 2000). Resolution of SC data was 0.01 μSiemens (μS).

The recording took place in a sound-attenuated, electrically shielded, air-conditioned and dimly illuminated room where participants sat in a comfortable chair. Air-conditioning maintained a constant temperature of 23 °C and relative humidity of the atmosphere of around 40%.

### Dependent variables

Dependent variables were: (1) the SCR strength and (2) specific values of the HR response. The HR response was split into separate intervals (dependent variables) to get these specific values.

As the *SCR* is a unidirectional response, its strength was measured in a conventional manner as amplitude. Thus, the difference between the minimum (occurring in a latency window of 1 to 3 s subsequent to stimulus onset) and the maximum of the SC increment (in the post-stimulus time frame of 1 to 9 s) was calculated. If no SCR occurred in response to a stimulus, an amplitude value of zero entered the subsequent statistical analyses. This was possible without serious consequences because none of the participants was a SC non-responder, i.e., none of them reacted to the first three or to all six numerals with less than one SCR.

According to the presumable component structure of the *HR response*, various predetermined time segments were considered for the determination of components. *Measuring values of change* (HR in Δbpm) were calculated for these segments because the response was defined as HR change (after stimulus onset) in relation to a baseline (HR in the last second prior to stimulus onset). The following measured values were collected: the mean value for the seconds 1–2 (HR_1-2_: as an indication of a first component, the primary bradycardia), 3–4 (HR_3-4_: as an indication of a second component which might reflect, for instance, an automatic call for processing, central processing like memory search, and resource competition between central and perceptual processing demands), and 5–8 (HR_5-8_: as an indication of a third component which might reflect particularly anticipatory orienting activity); as well as the HR difference of the seconds 7–8 minus 3–4 (HRd). The latter variable has been considered because it may be assumed to reflect pretty well the compound effects of our manipulation of signal value in one key variable. In addition, the HR response was measured as an average value across the seconds 1–4 after stimulus onset (HR_1–4_). This is a traditional time slot for measuring a novelty-dependent HR slowing (Zimmer, 2002), which we call “novelty deceleration”.

### Data analysis and statistical evaluation

*Response habituation* was analysed in consideration of all repetition trials (trials 1–6) and, additionally, for reasons of data smoothing, by means of 3 trial blocks consisting of 2 consecutive repetition trials each.

*Response recovery* was measured as the difference between the response on the test trial (trial 7) and the average response of the reference trials – the second half of the repetition trials (trials 4–6).

A repeated-measures effect over the 6 trials and over the 3 trial blocks was additionally decomposed into its linear and its quadratic orthogonal polynomial components. These trends were calculated to account for theoretical assumptions linking the CNS process of habituation to a negative exponential function (cf., e.g., Vossel & Zimmer, 1989b, p. 142). Accordingly, a significant repeated-measures effect attributable to a decrease in response strength that occurs together with a significant linear and quadratic trend across the trials or blocks can be accepted as indicative of an exponential function typical of habituation.

For the repeated-measures factors *trial* or *block*, the probability *p* of alpha error was readjusted using the Greenhouse–Geisser epsilon correction procedure. For reasons of simplicity, the original degrees of freedom are, however, presented, but along with the respective epsilon value (ε) and the significance level reached by the readjusted *p* value.

## Results

### The heart rate response and its component structure

**Fig. 2:**
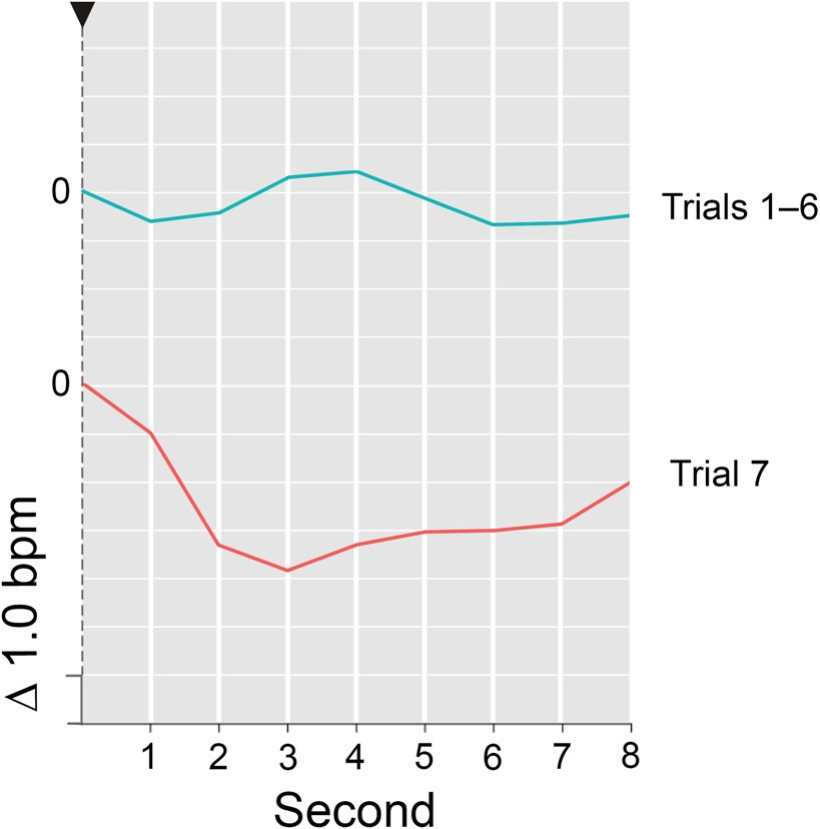
HR response (unit: Δbpm) throughout eight post-stimulus seconds (seconds subsequent to stimulus onset), averaged across participants (144) and presented for the test trial (7) and the average repetition trial (mean across 6 trials). Fixed timeframe for the reference measurement of the stimulus-induced HR response is the last second prior to stimulus onset (baseline value = 0). An arrow represents the moment of stimulus onset. The zero reference for the respective HR response is exactly the point where its curve intersects the vertical line. *Please note*, intersection of the various HR response curves with the vertical line does not reflect their actual baseline value—the respective HR value for the second before stimulus onset. Instead, the position of the curves on the vertical line was arbitrarily set for clarity of presentation

**Fig. 3:**
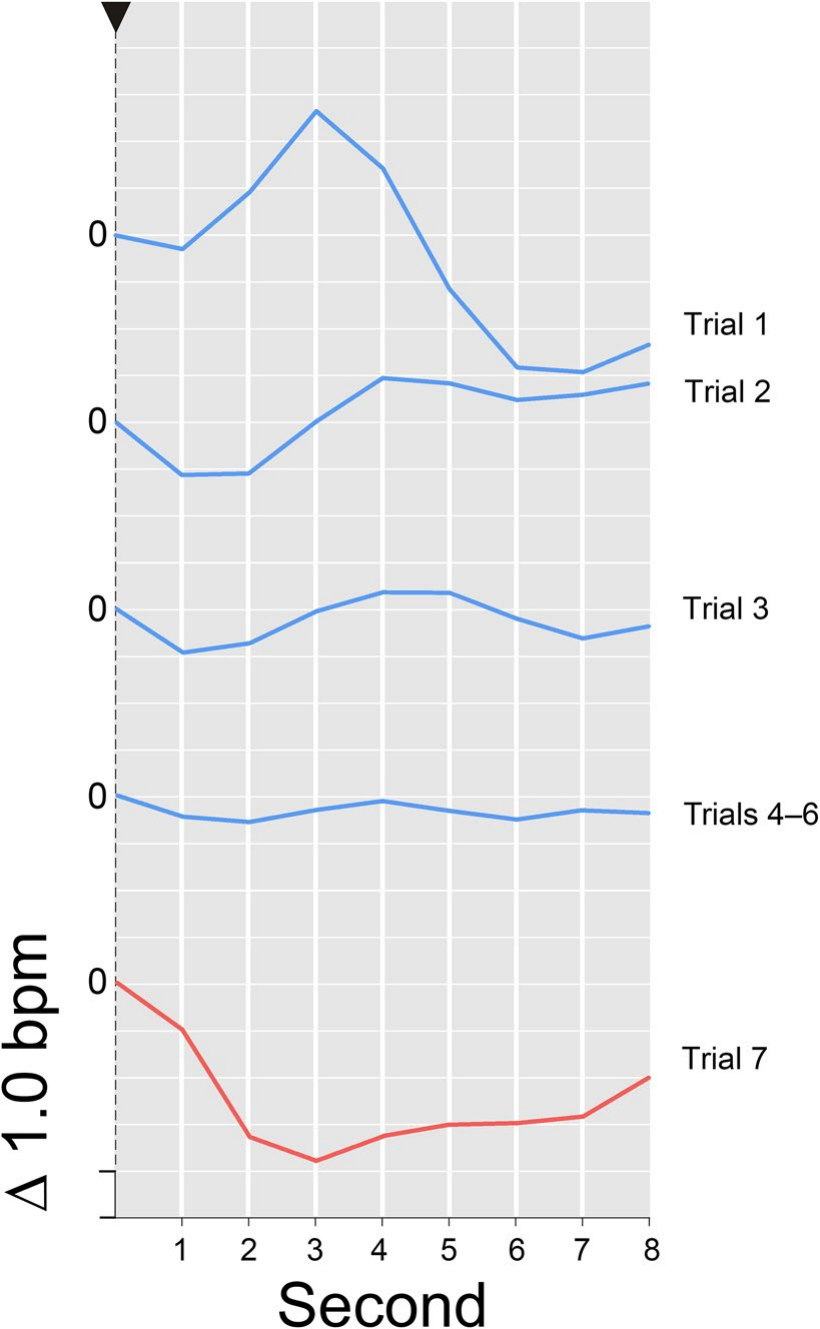
Time course of the HR response (in Δbpm), averaged across participants and presented as a function of post-stimulus seconds and trials (1, 2, 3, 4–6, 7). Stimulus onset is represented by an arrow. The zero reference for the respective HR response is exactly the point where its curve intersects the vertical axis. *Please note*, intersection of the various HR response curves with the vertical line does not reflect their actual baseline value—the respective HR value for the second before stimulus onset. Instead, the position of the curves on the vertical line was arbitrarily set for clarity of presentation

The average *HR response* across the habituation trials did not prove to be the uniform slowing that Zimmer (2002) found for similar stimuli and that is usually interpreted as an indication of involuntary orienting (e.g., Graham & Clifton, 1966; Turpin, 1983). Instead, a *polyphase response* emerged. It consisted of a transitory deceleration, quickly followed by an acceleration and another but longer-lasting deceleration (cf. Fig. 2). This pattern was particularly pronounced in response to the first stimulus (cf. Fig. 3).

Following the novel change in the test trial, the single-phase *HR response* fully appeared. It consisted of a *pronounced and persisting heartbeat slowdown* peaking in second 3 (cf. Fig. 2).

### Response habituation

**Fig. 4:**
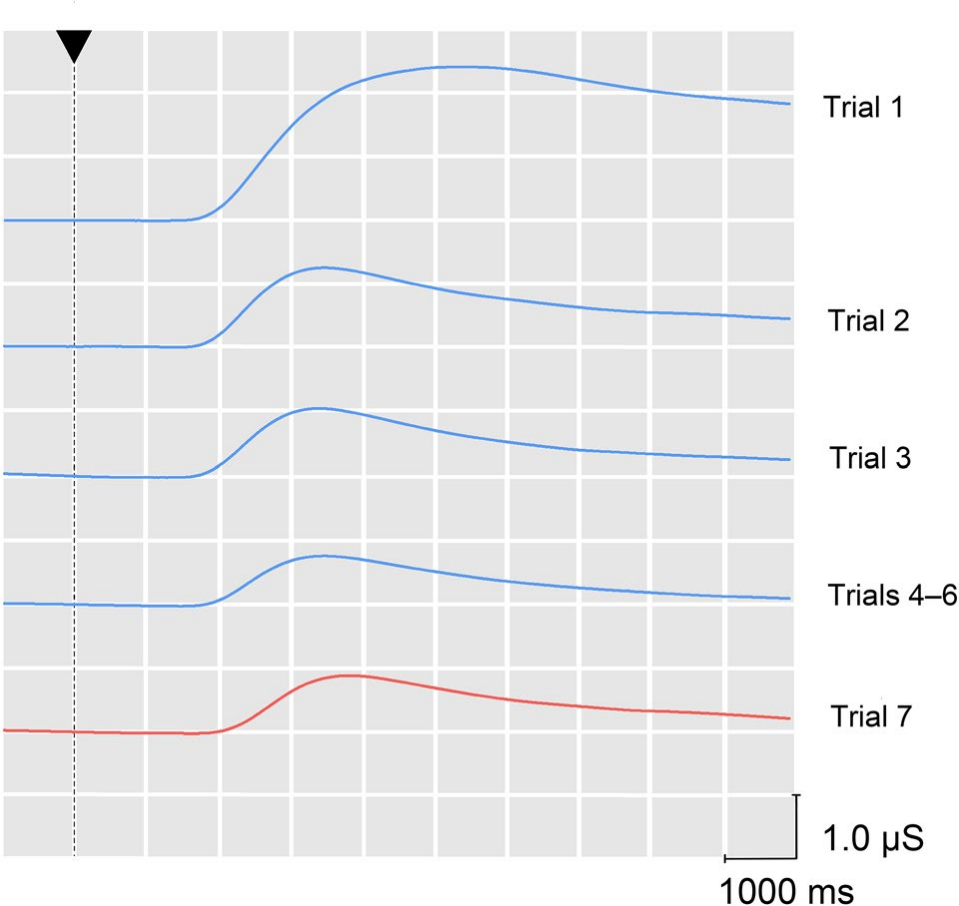
Event-related electrodermal activity depending on the repetition trials 1, 2, 3, 4–6 and the test trial (7). Point in time of stimulus onset is marked with an arrow. *Please note*, intersection of the event-related electrodermal activity with the vertical line does not reflect the actual level of electrodermal activity at the time of stimulus onset. Instead, the intersection point was arbitrarily set for clarity of presentation

**Fig. 5:**
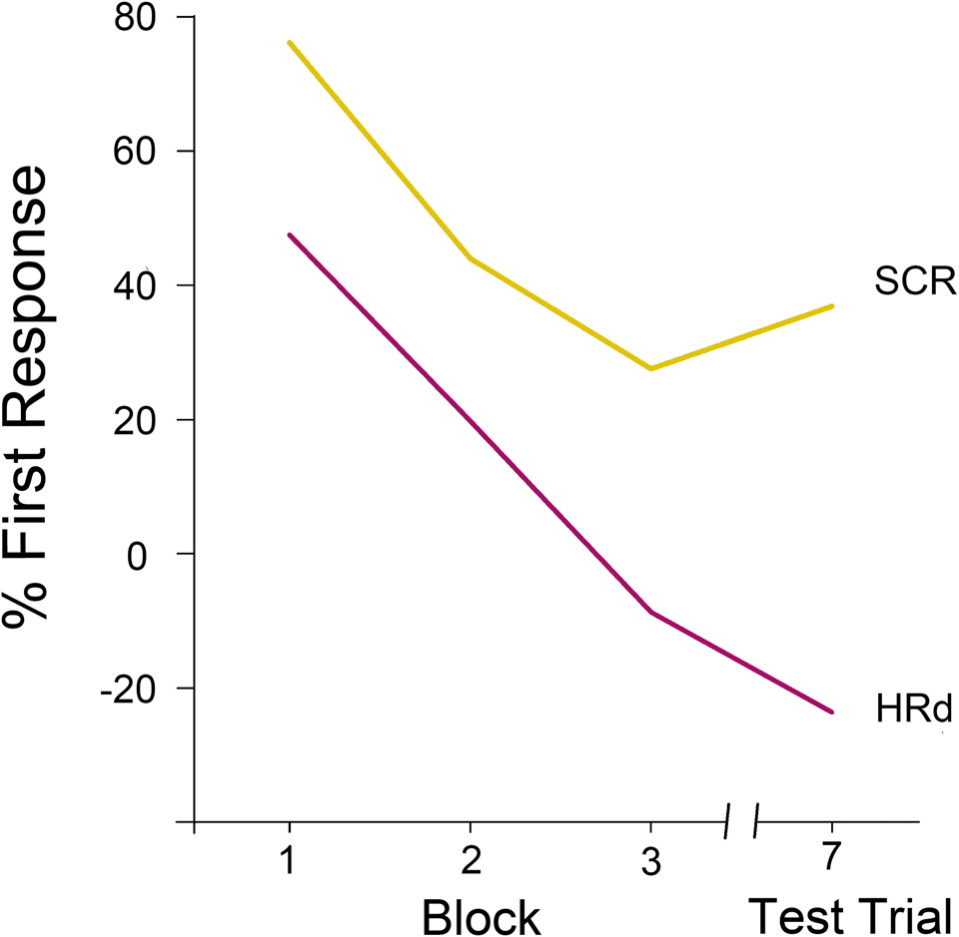
Graphic illustration of the effects of repetition and the change on two dependent variables (SCR and HRd). The effect of repetition is depicted across three blocks of two trials at a time. Data are expressed as a percentage of the respective first response

In regards to *response habituation*, i.e., regarding a systematic (usually exponentially) response decrement with repeated stimulation, which must be considered the *first criterion* of CNS habituation, the *SCR* behaved in line with theories of involuntary (novelty-dependent) orienting (cf. Tables 1 and 2). Typical of response habituation, the SCR showed a significant and almost exponential decline in response strength across the 6 (conceptual) repetition trials (cf. Fig. 4) and across the 3 blocks of these trials (cf. Fig. 5).

**Table 1:** F-ratios along with the Greenhouse–Geisser epsilon values (ε) for all main effects of interest

	Repetition				Change	Signal value
	Trial 1–6		Block 1–3			
	F_5,715_	(ε)	F_2,286_	(ε)	F_1,142_	F_1,142_
SCR	101.27**^1,*2*^	(0.390)	115.13**^1,2^	(0.672)	4.56*	0.11
HR_1–2_	2.62*	(0.838)	0.78	(0.847)	13.91**	0.55
HR_3–4_	3.43**^1^	(0.815)	5.69**^1^	(0.790)	49.45**	0.64
HR_1–4_	2.93*^1^	(0.835)	3.15^(^*^)1^	(0.814)	37.40**	0.76
HR_5–8_	4.44**^(1),2^	(0.830)	0.85	(0.851)	48.25**	1.05
HRd	23.65**^1,*2*^	(0.858)	24.91**^1^	(0.921)	10.07**	4.94*

**p < 0.01; *p < 0.05; ^(^*^)^ 0.05 < *p* < 0.10

^1^Repetition effect apparent in a linear trend (*p* < 0.05; italic type: *p* < 0.01; in parentheses: 0.05 < *p* < 0.10)

^2^Repetition effect apparent in a quadratic trend (*p* < 0.05; italic type: *p* < 0.01)

**Table 2:** SCR (in μS) and HR response components (in Δbpm) as a function of repetition (trials T1-T6), change (on 7th trial) and signal value (modes of orienting)

							Change	Signal value*
	T1	T2	T3	T4	T5	T6	T7	
SCR	2.700	1.407	1.192	1.178	0.857	0.625	0.992	0.027
HR_1–2_	0.301	− 1.137	− 0.853	0.125	− 0.412	− 1.271	− 2.164	0.233
HR_3–4_	2.042	0.459	0.144	0.248	− 0.577	− 0.353	− 3.569	0.321
HR_1–4_	1.172	− 0.339	− 0.355	0.186	− 0.494	− 0.812	− 2.866	0.277
HR_5–8_	− 2.318	0.672	− 0.217	− 0.704	− 0.127	− 0.343	− 2.745	− 0.371
HRd	− 4.680	0.244	− 0.645	− 1.196	0.561	0.267	1.111	− 0.701

*The depicted measurement is a difference value (response to targets minus response to non-targets)

In contrast to these SCR results, neither the repeated-measures factor *trial* nor the repeated-measures factor *block* resulted in a systematic reduction of a novelty-dependent HR deceleration in the traditional 4-s time slot. Analyses of response components, however, uncovered some informative results (cf. Fig. 3). These are illustrated below as changes across blocks (cf. Table 1). Across the 3 blocks the HR_3–4_, showed an initial acceleration, which later on turned into a deceleration (+ 1.251, + 0.196, − 0.465 Δbpm). The HRd (cf. Fig. 5), which is assumed to reflect the compound effects of our manipulation of signal value better than any single acceleration or deceleration measure alone, showed a corresponding and even stronger effect (− 2.218, − 0.920, + 0.414 Δbpm). Both effects were due to a significant (*p* < 0.01) linear trend. For comparison, neither the HR_1–4_, (+ 0.416, − 0.084, − 0.653 Δbpm) nor the HR_1–2_, (− 0.418, − 0.364, − 0.841 Δbpm) showed a comparable decrease in HR.

#### Response habituation—in consideration of task instruction

Both the SCR and the HR response failed to reflect significant effects of task instruction (the within-subjects manipulation of signal value) on the course of their response habituation.

### Effects of task instruction and target positioning

**Fig. 6:**
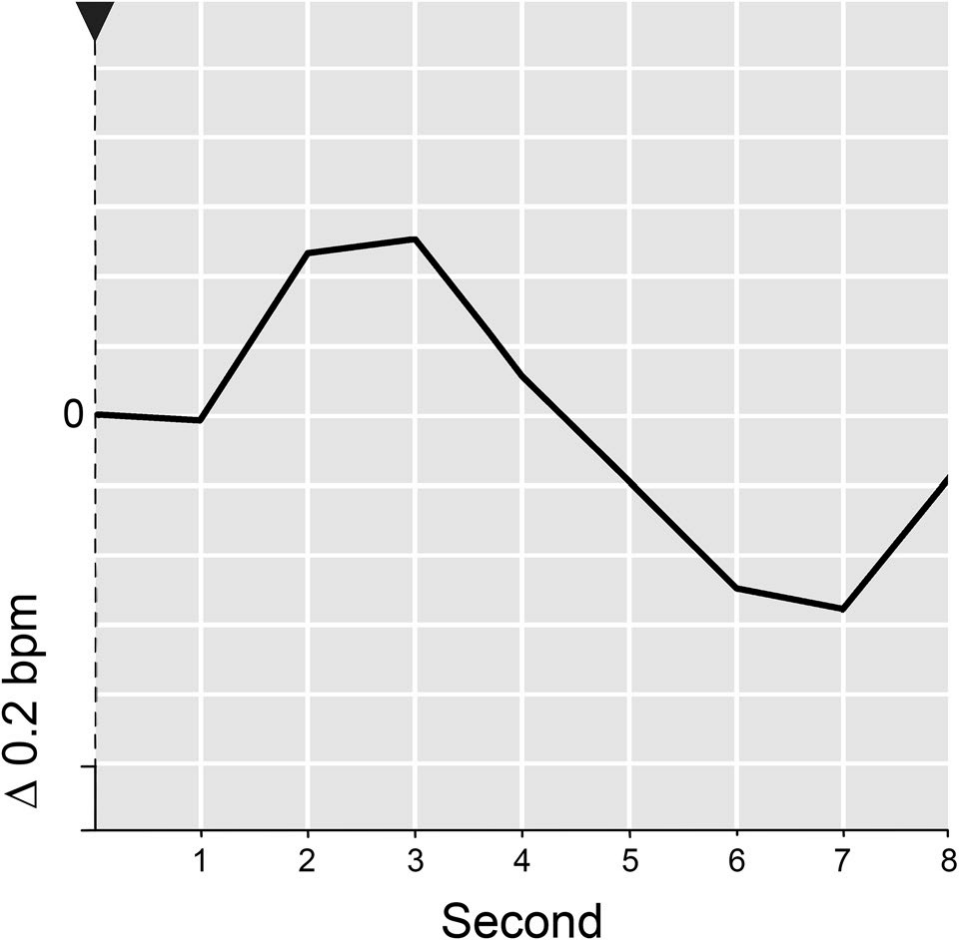
Net effect of signal value on the HR response—average difference between responses to targets and non-targets. *Please note*, the intersection of the HR response curve with the vertical line does not reflect the actual baseline value—the HR value for the second before stimulus onset. Instead, the position of the curve on the vertical line was arbitrarily set for clarity of presentation

**Fig. 7:**
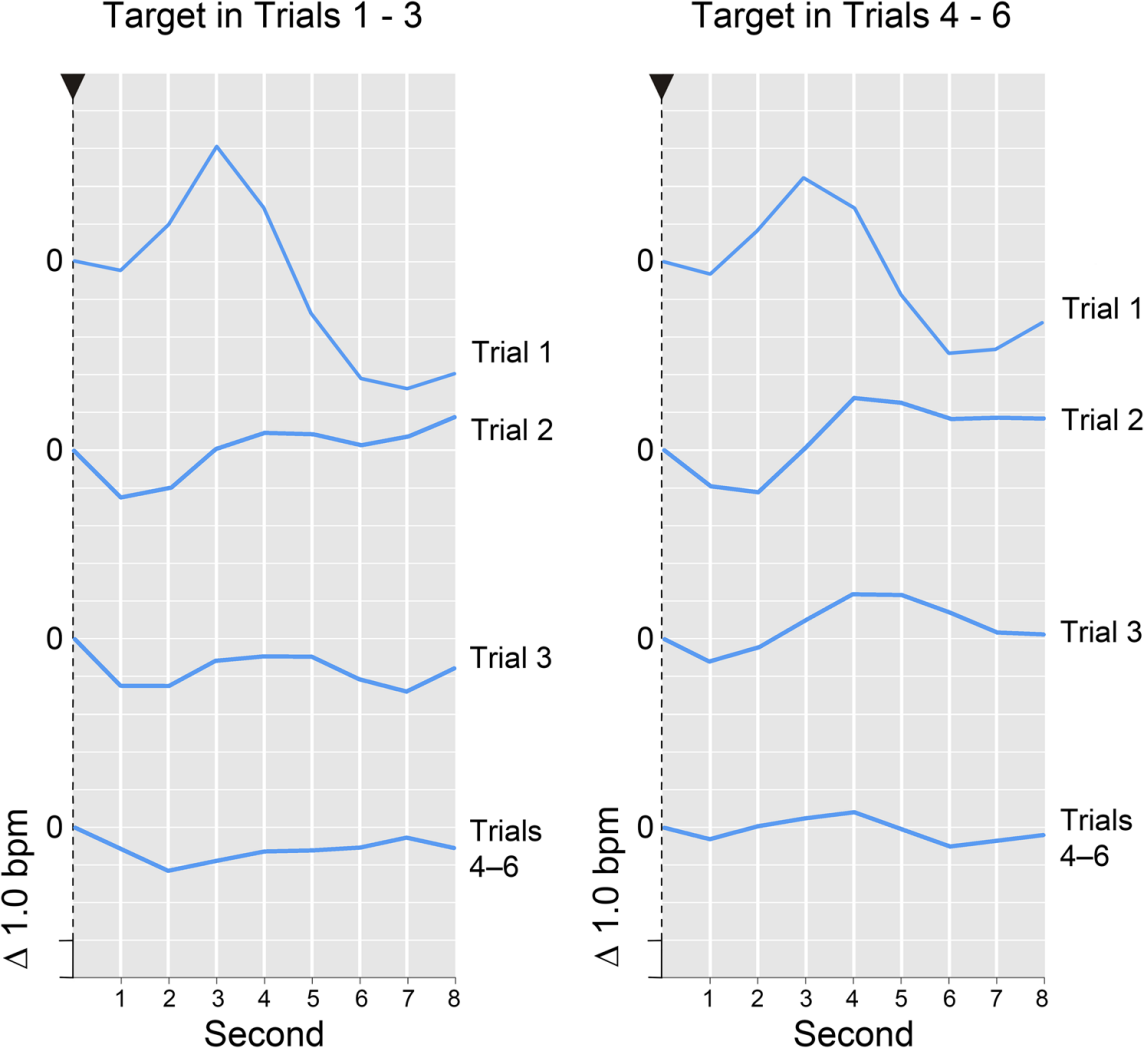
HR response (in Δbpm) as a function of target positioning (target in trials 1–3 vs. 4–6) and repetition (trials 1, 2, 3, 4–6). Stimulus onset is represented by an arrow. The zero reference for the respective HR response is exactly the point where its curve intersects the vertical axis. *Please note*, the intersection of the various HR response curves with the vertical line does not reflect their actual baseline value—the respective HR value for the second before stimulus onset. Instead, the position of the curves on the vertical line was arbitrarily set for clarity of presentation

In almost all dependent variables examined, the effects of task instruction (the two-step manipulation of signal value) did not reach the level of significance. Only in the *HRd,* a principal effect of task instruction emerged (cf. Table 1 and Fig. 6), and, yet more importantly, this within-subjects effect was dependent upon the between-subjects factor *target positioning* (*F*[1,142] = 24.86, *p* < 0.01; cf. with Fig. 7).

Close inspection of the HRd and especially its component parts revealed that the higher signal value of the targets as compared to the non-targets slightly—albeit non-significantly—strengthened the acceleration in second 3–4 (+ 0.488 vs + 0.167 Δbpm) and the deceleration in second 7–8 (− 0.771 vs − 0.391 Δbpm). This, however, turned out to be predominantly true when the targets occurred in the first half and not in the second half of the habituation trials (HR_3–4_: + 1.430 vs − 0.788 Δbpm, *F*[1,142] = 7.59, *p* < 0.01; HR_7–8_: − 0.842 vs + 0.083 Δbpm, *F*[1,142] = 1.43, n.s.).

### Response recovery

In regards to the *second criterion* of CNS habituation as a selective CNS inhibition process, namely selectivity demonstrated by *response recovery* to a novel change in stimulation, recovery effects were found in both response systems examined (cf. Tables 1, 2 and Figs. 3, 4, 5).

While the SCR increased, the statistical effects of the changed stimulation on the cardiac response components stemmed from a pronounced and persisting slowing of the heart rate that contrasts with a weak and rather flat HR response in the reference trials (cf. Fig. 3).

#### Response recovery—in consideration of task instruction

Recovery of the SCR and of almost all HR response components (HR_1–4_, HR_1–2_, HR_3–4_, HR_5–8_) was not significantly dependent upon signal value—the task regarding the stimuli of the reference trials.

In contrast, recovery of the HRd was significantly dependent of the task (*F*[1,142] = 4.75, *p* < 0.05). The recovery effect was to a great extent due to the task of voluntary orienting (HRd in response to the novel stimulus: + 1.470 Δbpm, in comparison with the reference measurement to targets: − 0.611 Δbpm; HRd in response to the novel stimulus: + 0.752 Δbpm, in comparison with the reference measurement to non-targets: + 0.366 Δbpm – difference: 2.081 vs 0.386 Δbpm).

## Discussion

The present study was undertaken to address the question of whether two prominent peripheral responses similarly reflect the brain activity of involuntary and voluntary orienting.

### Skin conductance response

The SCR findings reported here provide evidence of their exemplariness in the context of Sokolov’s (1963) *involuntary OR*, because they unambiguously indicated, regardless of the task at hand, *novelty processing* and *habituation* in the human brain. That is to say, SCR amplitude reflected—as expected for a valid indicator of involuntary orienting—actual as well as contextual novelty. Moreover, it declined almost exponentially in response to a conceptual repetition—consisting of six spoken numerals which were, at least in the beginning, contextually novel stimuli. Typical for the CNS process of habituation, the decline turned out to be selective, because it was followed by a recovered SCR to a changed stimulation. In the present case, the occurrence of a unique and actually novel sound brought about this change.

Although the SCR is known for its susceptibility to stimulus significance (e.g., Bernstein, 1969, 1979; Dawson et al., 1989; Pendery & Maltzman, 1977) or voluntary orienting (Maltzman, 1979, 1990) its amplitude stayed unresponsive to our experimental manipulation of *signal value*—implemented by a task instruction that prompted participants to give attention actively and voluntarily to some of the numerals (targets) as well as their immediate consequences and to disregard passively the others (non-targets). In regards to the SCR, the realised two-step manipulation of signal value was consequently either too weak or in other respects ineffective. Becker and Shapiro (1980) likewise concluded from a similar SCR result, “that either simply attending to a stimulus does not make that stimulus significant to any large degree or that stimulus significance only affects the OR in a limited range of conditions” (p. 389). Be that as it may, key factors of voluntary orienting, notably effort (Kahneman, 1973; Verbaten, 1983), personal relevance (Zimmer, 2000) or, more widely, motivational activation (Bradley, 2009), are known for their considerable impact on the SCR but were deliberately not manipulated to any great extent by our instruction.

### Heart rate response

Unlike the SCR, the HR response clearly reflected both involuntary and voluntary orienting, although, as expected, not in a similar manner.

In response to the task-irrelevant and indeed novel stimulus, a pronounced and *long-running HR deceleration* appeared—as expected in hypothesis A–I. This deceleration is quite typical for an HR response to a unique and unconditioned change stimulus in a repetition-change paradigm (cf. Bohlin et al., 1981; Vossel & Zimmer, 1989a, 1992; Zimmer, 2002). It may thus be regarded in line with the tenor of prior publications on the OR (cf., e.g., Turpin, 1983) as tentative evidence of *involuntary orienting*.

However, it is important to note that this putative *“novelty deceleration”* does not prove a true recovery of a previous and, as a result of habituation, declined novelty-dependent HR deceleration. In fact, the mean HR response on the preceding conceptual repetition trials (cf. Fig. 2), as well as the HR response to contextual novelty (cf. Fig. 3), was definitely not a uniform deceleration, but rather a *polyphase response* resembling the HR change in the foreperiod of signalled reaction time paradigms (Bohlin & Kjellberg, 1979). The response consisted of three constituent parts: a transitory and non-habituating deceleration, most likely and solely representing the stimulus-driven primary bradycardia (cf. Graham, 1992; Lacey & Lacey, 1980), followed by rapid acceleration and an ensuing deceleration. According to Bohlin and Kjellberg (1979) evidence suggests that these are relatively independent HR response components at least in signalled reaction time paradigms.

Thus, the assumption that a novelty-dependent HR deceleration also occurs inevitably contingent on contextual novelty cannot be verified by the present data. Primarily, it cannot account for the fact that a heartbeat-accelerating response component was particularly strong in early trials, was not displaced by a noticeable HR deceleration in early non-target trials, and also showed evidence of response habituation.

Yet, this is not a totally surprising result or one that could challenge stringently the common hypothesis of an HR deceleration occurring along with involuntary orienting. It is not a surprising result because the polyphase response characteristic was expected as a result of *voluntary orienting* (see hypothesis A-II).

As a whole the results are in line with the expectation that the HR is not only responding to involuntary or passive processes—as reflected by the primary bradycardia and the novelty-dependent HR deceleration – but also to other factors such as task-oriented voluntary and thus active adjustments of attention. These factors might be in large part responsible for the second and the third component of the polyphase response and, in general, they may vary to a considerable degree depending on the specific processing requirements of the task at hand (cf. with the effect of signal value on the response variable HRd). Such factors may be as well determinants in case of events which have an unclear task-relevance to participants—as in the standard version of the repetition-change paradigm.^1^

Anyway, mere listening to contextually novel but in other respects highly familiar stimuli probably cannot be held responsible for the polyphase response characteristic, especially since Zimmer (2002) found a clear HR deceleration to orienting stimuli which differed from the present ones only in task demands. In this previous HR study, the participants were only asked to listen to a train of verbal stimuli and to relax passively, whereas in the present study they were additionally requested to regard some of these verbal stimuli as signals for a definite task. By instruction, they were invited to find out whether these target stimuli were spoken by a female voice and also contaminated or followed by a noise. In short, this means that the participants had to compare *each* verbal stimulus with the targets’ memory representation. In case of target detection, they moreover had to identify the speaker’s gender and to search for a definite event, a noise superimposing or following the target.

On the assumption that an HR response is in the end a consequence of various processing operations, the manifestation of a specific HR component may, at the individual level, even result from the rivalry of opposing higher-level CNS processes which are involved in a temporary competition for limited perceptual-central resources (Wickens, 1980, 1984). For this reason, the HR acceleration found may be the result of task-based *central* processing requirements which temporarily dominate *perceptual* requirements. An acceleration is supposed to depend on, for example, demands upon controlled and elaborated memory processes (e.g., Jennings et al., 1990; Simons et al., 1998), whereas a deceleration is supposed to depend on, especially, demands upon sensory information reception (e.g., Graham & Hackley, 1991; Jennings et al., 1990).

Based on the differential outcomes depending on the experimental manipulation of signal value, the HR response can be supposed as more prone to voluntary influences than the SCR. This distinct susceptibility to voluntary orienting could be the main reason for the contradictory opinions on the question whether a measured HR response to novelty can be considered as a valid indicator of the involuntary OR (cf., e.g., Barry, 1986; Simons et al., 1987, 1998; Turpin et al., 1999; Vossel & Zimmer, 1989a).

Nonetheless, the present study showed that an HR deceleration is able to reflect the involuntary OR if but only if the orienting stimulus is a unique and highly novel change in stimulation. This (uniform) novelty-dependent deceleration has not currently been found in response to contextual novelty, although a novelty-based OR—as indicated by the SCR—occurred in response to both phenomena, actual *and* contextual novelty.

All in all, the present HR results are consistent with Sokolov’s (1990) assumption that the “significance contribution to the OR is achieved via frontal lobe activation of voluntary attention” (p. 99). Indeed, our task instruction regarding target stimuli was characterized by targeting voluntarily controlled intentions to attend some specific stimuli and their immediate consequences—something that is typical of lifelike voluntary orienting. The HR results are also compatible with the findings of Simons et al. (1998) that task-based higher mental functions, such as central processing and decision-making, induce HR acceleration. Different patterns of HR response might thus reflect functionally different control modes of selective information processing.

## Summary

To put it in a nutshell, the SCR once again has proven its prominent position among the indicators of the human involuntary OR. Regardless of the task at hand, the SCR reflected involuntary orienting, its habituation in consequence of conceptual repetition and its recovery as a result of a novel change in stimulation. Unlike skin conductance, the heart rate showed a non-uniform pattern of response and turned out to be susceptible to both involuntary and voluntary orienting. While the HR response to the last orienting stimulus, actual novelty, was a clear-cut deceleration, the HR response to the first orienting stimulus, contextual novelty, was neither a deceleration nor an acceleration alone but a polyphase response being sensitive to conceptual repetition and the requirements of a voluntary orienting. Thus, even if a slowing of the heart rate, were under some circumstances a reliable accompanying phenomenon of an involuntary OR, it would still be an invalid or at least restricted indicator of this OR because of its striking susceptibility to voluntary orienting.

## Footnotes

1 Additional factors may be arousal, arousal inhibition, and an increasing inhibition of competing actions. For the relation of the OR, arousal and a brief, rapid-onset – onset latency < 2 s – HR acceleration peaking in post-stimulus second 3, see Graham and Hackley (1991). For the relation of arousal inhibition and HR deceleration see klein Selle et al., (2016, 2017). For the relation between the late decelerative HR component and an increasing inhibition of competing actions during response selection see Jennings and Van der Molen (2002, 2005).
